# Temporary Uterine Artery Clamping During Laparoscopic Myomectomy and Perioperative Hemoglobin Decline: A Retrospective Single-Center Comparative Study

**DOI:** 10.3390/biomedicines14102398

**Published:** 2026-10-20

**Authors:** Engin Korkmazer, Rulin Deniz, Alihan Tigli, Oguzhan Karakoc, Yasemin Ercan Degirmenci, Nazli Sener, Guzide Ece Akinci, Yasemin Albayram, Esra Soylemez, Dilsad Sonmez Ozgur, Mehmet Ali Aydin, Yakup Baykus

**Affiliations:** 1 Department of Obstetrics and Gynecology, Private Practice,16000 Bursa, Turkey; enginkorkmazer@gmail.com; 2 Department of Obstetrics and Gynaecology, Faculty of Medicine, Bandırma Onyedi Eylül University, 10200 Bandırma, Turkey; rdeniz@bandirma.edu.tr (R.D.); atigli@bandirma.edu.tr (A.T.); ydegirmenci@bandirma.edu.tr (Y.E.D.); nsener@bandirma.edu.tr (N.S.); 3 Gynecology and Obstetrics Clinic, Bandırma Training and Research Hospital, 10200 Bandırma, Turkey; oguzhankarakoc1986@hotmail.com (O.K.); yasemingalbayram@gmail.com (Y.A.); 4 Gynecology and Obstetrics Clinic, Şehit Prof. Dr. İlhan Varank Sancaktepe Training and Research Hospital, 34785 Istanbul, Turkey; geceakinci@gmail.com; 5 Department of Obstetrics and Gynecology, Private Practice,10200 Bandırma, Turkey; esraa.soylemezz@gmail.com; 6 Gynecology and Obstetrics Clinic İnegöl State Hospital, İnegöl, 16400 Bursa, Turkey; dilsadsnmz@gmail.com; 7 Ministry of Health of the Republic of Turkey, Office of the Chief of Staff, 06434 Ankara, Turkey; mehmetali.aydin@saglik.gov.tr

**Keywords:** laparoscopic myomectomy, temporary clamping of the uterine artery, perioperative decrease in hemoglobin, absolute decrease in hemoglobin, percentage decrease in hemoglobin, uterine leiomyoma

## Abstract

**Background/Objectives**: The aim of this study was to evaluate the adjusted association of temporary uterine artery clamping with the absolute and percentage decreases in hemoglobin during the perioperative period in women undergoing laparoscopic myomectomy, and to evaluate this association after multivariable adjustment for patient and leiomyoma characteristics. **Methods**: This single-center, retrospective, comparative observational study included 118 patients who underwent elective laparoscopic myomectomy for uterine leiomyomas between January 2021 and January 2026 (65 with temporary uterine artery clamping and 53 controls). The decision to perform clamping was based on the surgeon’s intraoperative assessment. The primary outcome measures were defined as absolute hemoglobin drop and percentage hemoglobin drop. The adjusted association of clamping with these measures was examined using multivariable linear regression models simultaneously adjusting for age, body mass index (BMI), and tumor burden (evaluated as dominant diameter in Model 1 and specimen weight in Model 2), with baseline hemoglobin additionally adjusted in absolute decline models. **Results**: No significant differences were found between the two groups in terms of baseline clinical and leiomyoma characteristics (age, BMI, number of pregnancies, preoperative hemoglobin level, leiomyoma size, and weight) (all *p* > 0.05). Although the difference in postoperative hemoglobin levels did not reach statistical significance (9.59 ± 1.70 vs. 10.01 ± 1.45 g/dL; *p* = 0.153), both the absolute hemoglobin decline (controls vs. clamping: 2.20 ± 0.90 vs. 1.83 ± 0.88 g/dL; *p* = 0.027) and the percentage hemoglobin decline (controls vs. clamping: 18.84 ± 7.77% vs. 15.24 ± 6.61%; *p* = 0.008) were significantly lower in the clamping group. Evaluated as a composite safety outcome across all 134 surgical patients (transfusion or conversion to laparotomy), procedural safety events occurred in 18.5% (12/65) of controls compared with 5.8% (4/69) of clamping cases, demonstrating a statistically significant reduction in composite major surgical morbidity in the clamping cohort (Fisher’s exact test *p* = 0.033; Relative Risk = 0.31, 95% CI: 0.11 to 0.92). In both the primary and sensitivity multivariable models, temporary uterine artery clamping demonstrated a significant adjusted association with lower values of approximately 0.38 g/dL in absolute hemoglobin decline (*p* = 0.015–0.016) and approximately 3.6 percentage points in percentage hemoglobin decline (*p* = 0.007–0.008). Furthermore, while the preoperative hemoglobin level was independently and positively associated with the absolute decrease in hemoglobin (*p* < 0.001 in both absolute decline models), age, BMI, leiomyoma size, and weight showed no independent association. **Conclusions**: Temporary uterine artery clamping demonstrated a significant multivariable adjusted association with a modestly attenuated perioperative hemoglobin decline during laparoscopic myomectomy. The finding that static postoperative hemoglobin levels alone did not differ significantly between groups underscores the importance of using dynamic hemoglobin parameters in the assessment of surgical blood loss. Although the retrospective design limits causal inferences, temporary uterine artery clamping appears to be a feasible surgical adjunct associated with reduced perioperative hemoglobin decline in experienced hands; however, prospective trials evaluating operative duration, clamping intervals, and long-term reproductive safety are warranted before broader clinical safety can be definitively established. Temporary uterine artery clamping demonstrated a significant multivariable adjusted association with a modest attenuation in perioperative hemoglobin decline (approximately 0.38 g/dL) during laparoscopic myomectomy, whereas static postoperative hemoglobin levels alone did not differ significantly between groups. Given the modest clinical magnitude of this difference, the lack of a significant difference in standalone transfusion rates, and the potential for residual confounding by indication in this retrospective cohort, temporary clamping should be considered a feasible adjunctive measure in experienced hands rather than a definitive routine intervention, and prospective randomized trials are warranted.

## 1. Introduction

Uterine leiomyomas are among the most common benign tumors of the female reproductive system and primarily affect women of reproductive age [1]. The prevalence of leiomyomas has been reported to range widely from 4.5% to 68.6%, depending on the type of study, diagnostic method, and the racial/ethnic characteristics of the study population; data based on ultrasound screening indicate that leiomyomas are detected in approximately 70% of white women and more than 80% of women of African descent by age 50 [2]. Although a significant proportion of leiomyomas are asymptomatic, symptomatic cases may present with abnormal uterine bleeding, anemia, pelvic pain, compression symptoms, urinary or gastrointestinal complaints, infertility, and adverse pregnancy outcomes [2]. Clinical findings are largely determined by the number, size, and anatomical location of the leiomyomas, as well as their relationship to the endometrial cavity and the myometrium [3]. In Türkiye, although available data are limited, uterine leiomyomas are among the most common benign gynecologic conditions in reproductive-aged women; in the largest Turkish prevalence study, leiomyomas were detected by transvaginal ultrasound in 27.8% of symptomatic and 12.2% of asymptomatic women, with approximately half of all leiomyomas remaining asymptomatic [4]. However, the vast majority are managed expectantly or medically in primary and secondary care. Surgical intervention is concentrated in specialized tertiary centers and reserved for patients experiencing refractory abnormal uterine bleeding, pelvic pressure, chronic pain, or reproductive impairment actively seeking fertility preservation.

Myomectomy is a uterine-preserving surgical treatment option for symptomatic women who wish to preserve their fertility [1]. In appropriate patients, laparoscopic myomectomy has become an important minimally invasive surgical procedure because it is associated with less postoperative pain, a shorter hospital stay, faster recovery, and lower perioperative morbidity compared to the laparotomy approach [5]. In addition, current comparative evidence shows that laparoscopic myomectomy yields similar outcomes to laparotomy myomectomy in terms of pregnancy outcomes [6]. However, the laparoscopic approach can be technically challenging, particularly in cases of large, numerous, deeply intramural leiomyomas, or those with pronounced vascularization [7].

Intraoperative bleeding is one of the major surgical complications encountered during myomectomy [8]. The highly vascular nature of the myometrium and leiomyomas can lead to significant blood loss during myometrial incision, leiomyoma enucleation, and reconstruction of the uterine tissue. The localization and myometrial depth of leiomyomas are standardized according to the FIGO (International Federation of Gynecology and Obstetrics) classification, which includes eight subtypes and intermittent staging (hybrid forms) in cases where a leiomyoma extends through more than one layer [9]. The amount of bleeding is influenced not only by patient- and tumor-specific characteristics—such as the size, number, location, and weight of the leiomyomas—but also by the duration of the surgery and the hemostatic methods used [10]. Because these characteristics include location and myometrial depth, the FIGO type has been reported as an independent predictor of blood loss and hemoglobin decline during myomectomy in a study [11]. Excessive perioperative bleeding can not only impair the surgical field of view and lead to a longer operation time, but it can also negatively affect the recovery process by increasing the risk of postoperative anemia and the need for blood transfusions, and may contribute to perioperative morbidity [12,13]. For this reason, various pharmacological, mechanical, and surgical methods have been developed to reduce blood loss during myomectomy [14]. These include vasoconstrictor agents, antifibrinolytic therapies, uterine tourniquet application, vascular ligation, and temporary or permanent occlusion of the uterine arteries [15]. Although intramyometrial vasopressin injection is a commonly used pharmacological approach to reduce intraoperative bleeding during laparoscopic myomectomy, its potential cardiovascular side effects and the fact that it is not routinely available at every center highlight the need to evaluate mechanical and temporary vascular control techniques as effective and reliable alternatives.

Temporary uterine artery clamping is a surgical strategy aimed at reducing uterine perfusion during the hemorrhagic phase of surgery and restoring blood flow once leiomyoma enucleation and uterine repair are complete. This approach differs fundamentally from uterine artery embolization or permanent ligation procedures, in which the uterine arteries are permanently occluded; since the clamping is temporary, uterine blood flow is restored once the surgery is complete. In a systematic review and meta-analysis evaluating 12 studies involving a total of 1623 patients, it was reported that laparoscopic uterine artery occlusion significantly reduced intraoperative blood loss, postoperative hemoglobin decline, and transfusion rates; it also tended to shorten the length of hospital stay and may reduce the recurrence rate; however, it was emphasized that the findings should be interpreted with caution due to the moderate methodological quality of the studies and moderate-to-high heterogeneity in some outcomes [16]. In a prospective comparative study, intraoperative blood loss was found to be significantly lower in the group that underwent temporary uterine artery occlusion; however, there was no significant difference in operative time or postoperative hemoglobin levels compared to the control group that underwent standard laparoscopic myomectomy [17]. In a retrospective comparative study, blood loss was found to be significantly lower in the group that underwent uterine artery ligation; however, the duration of the procedure was approximately 15 min longer than in the group that did not undergo ligation [18]. This suggests that vascular control techniques and methods for assessing blood loss may play a role in the variability observed in the literature.

In addition to these event data, the effect of temporary uterine artery clamping on reproductive functions was also evaluated. In a systematic review and meta-analysis comparing patients who underwent clamping with those who did not, no significant difference was found in live birth and clinical pregnancy rates [19]. Similarly, no significant decline in anti-Müllerian hormone (AMH) and follicle-stimulating hormone (FSH) levels was observed during a six-month follow-up in women who underwent myomectomy with uterine artery occlusion; however, it was reported that early-stage changes in antral follicle count, ovarian volume, and ovarian stromal blood flow regressed during the follow-up period [20]. Although these data suggest, based on current short- and medium-term evidence, that temporary uterine artery clamping does not have a significant adverse effect on reproductive function, data regarding long-term outcomes are limited. Despite this heterogeneity in the current literature, the effect of temporary uterine artery clamping on perioperative hemoglobin changes—and whether this association persists after adjusting for clinical and leiomyoma-related characteristics—has not been sufficiently clarified.

Accurately and reliably measuring perioperative blood loss during laparoscopic myomectomy poses a significant methodological challenge. Calculations based on the volume of blood collected via the aspiration system and the irrigation fluid used may inaccurately reflect actual blood loss due to fluid dilution and the inability to fully account for blood accumulated on surgical instruments. Furthermore, it has been reported that hidden blood loss (HBL) occurring after myomectomy can account for a significant portion of total blood loss, and that assessing only visible blood loss may underestimate the actual loss [13]. For this reason, perioperative changes in hemoglobin levels are used as a complementary and more objective measure in the assessment of blood loss. However, changes in hemoglobin levels are an indirect indicator and may be influenced by factors such as perioperative fluid replacement, the timing of blood sample collection, and blood transfusion [21]. Evaluating the decrease in hemoglobin both as an absolute value and as a percentage change relative to the preoperative hemoglobin level may help account for differences in baseline hemoglobin levels and provide a more comprehensive picture of perioperative hematological changes.

The aim of this study is to evaluate the relationship between temporary uterine artery clamping and perioperative changes in hemoglobin levels in women undergoing laparoscopic myomectomy. To this end, the absolute and percentage decreases in hemoglobin levels were compared between patients who underwent temporary uterine artery clamping and those who did not; furthermore, the study aimed to evaluate the adjusted association between temporary uterine artery clamping and perioperative hemoglobin decline after multivariable adjustment for measured patient and leiomyoma characteristics. The hypothesis of the study is that the perioperative absolute and percentage decreases in hemoglobin will be lower in cases where temporary uterine artery clamping is performed.

## 2. Materials and Methods

### 2.1. Study Design and Patient Population

This study was conducted as a single-center, retrospective, comparative observational study. The study was reported in accordance with the Strengthening the Reporting of Observational Studies in Epidemiology (STROBE) guidelines for observational studies. The study population consisted of women who underwent elective laparoscopic myomectomy for uterine leiomyoma at the Gynecology and Obstetrics Clinic of Bursa Private Jimer Hospital between January 2021 and January 2026.

During this period, a total of 171 patients were retrospectively screened from clinical surgical records based on their surgery dates. The study included patients who underwent elective laparoscopic myomectomy for uterine leiomyomas, whose surgeries were completed laparoscopically, and for whom complete basic clinical and surgical data—including preoperative and postoperative hemoglobin levels—were available. All included patients were reproductive-aged women undergoing elective surgery for symptomatic leiomyomas with documented American Society of Anesthesiologists (ASA) Physical Status I or II. Patients with active hematological, renal, or hepatic disease, or documented coagulopathies, were excluded. Of the 171 screened patients, 37 were excluded preoperatively due to baseline non-surgical or medical factors: missing hemoglobin data (*n* = 13), concurrent major pelvic surgery (*n* = 11), documented coagulopathies (*n* = 5), and therapeutic anticoagulation (*n* = 8). Among the remaining 134 patients who commenced laparoscopic myomectomy (the surgical cohort: 65 controls and 69 temporary clamping cases), 16 experienced procedural safety events: 9 received allogeneic blood transfusions (7 control, 2 clamping) and 7 required conversion to open laparotomy (5 control, 2 clamping). Cross-referencing surgical and blood bank records confirmed mutually exclusive categorization without co-indexing (overlap = 0; none of the patients experienced both events simultaneously). In the clamping cohort, conversions were necessitated by severe retroperitoneal adhesions preventing vascular access before clamp placement could be performed. Patients receiving blood transfusions were excluded from continuous hemoglobin models to avoid biochemical confounding, while transfusions and conversions were evaluated as dedicated clinical safety outcomes across the 134 surgical cases.

**Figure 1:**
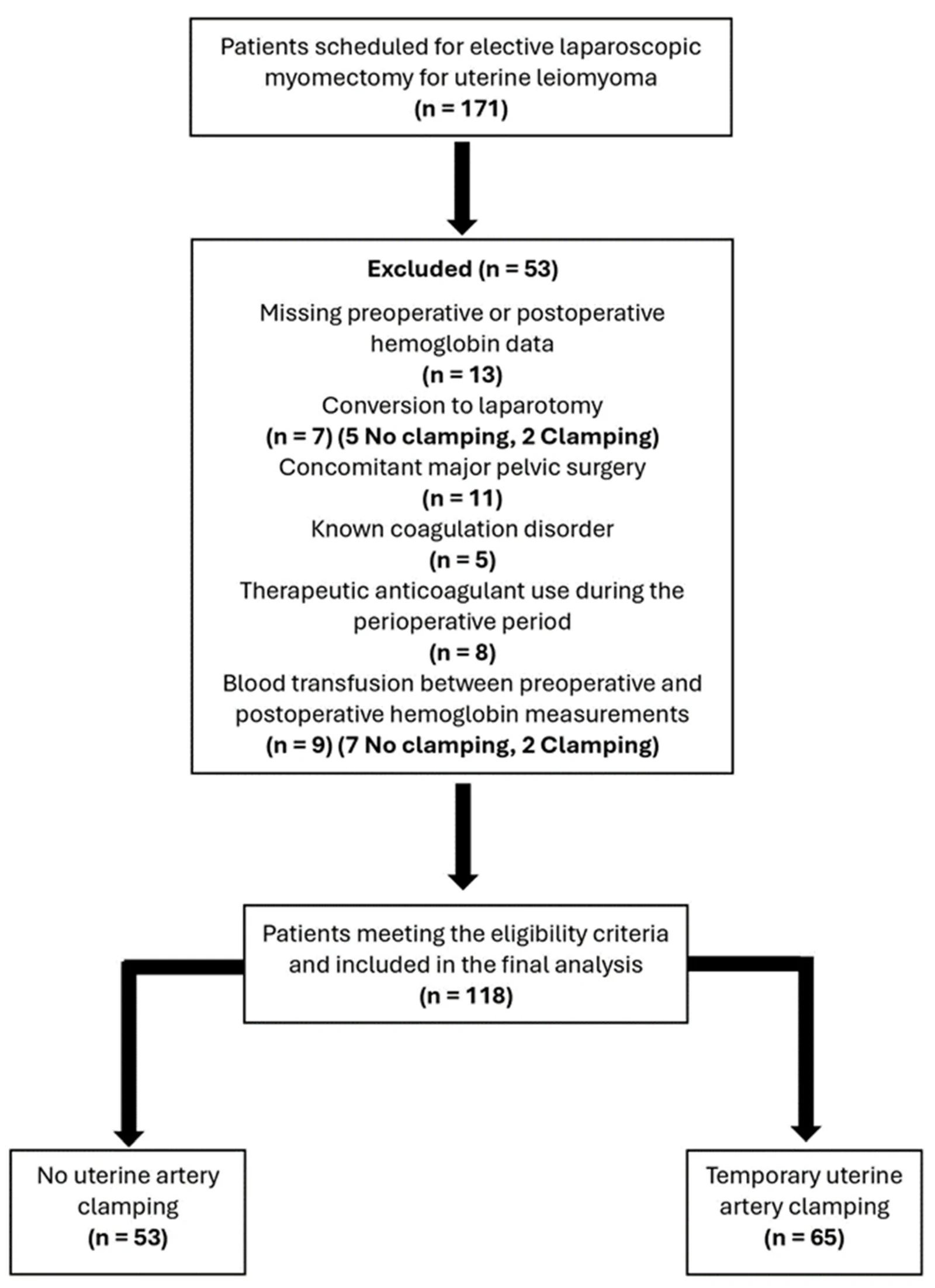
Flowchart of the Study Design.

A total of 118 patients who met the eligibility criteria were included in the primary hemoglobin decline analysis (65 with completed temporary uterine artery clamping and 53 controls). For procedural safety and surgical complication reporting, group allocation was governed strictly by the intention to clamp established upon initial diagnostic laparoscopic inspection (yielding an operative intention-to-treat cohort of 69 in the clamping arm and 65 in the control arm). The groups were not formed by randomization; no predefined protocol was used to determine clamping, and the decision to perform temporary uterine artery clamping was made for each patient based on the surgeon’s assessment at initial diagnostic laparoscopic inspection. Clamping was selectively indicated at this initial inspection in cases with pronounced myometrial vascularity, deep intramural extension, or anticipated reconstructive challenge, provided that the retroperitoneal anatomy appeared to permit safe dissection at that time. Surgical procedures were distributed consistently across the early (2021–2023: 31 clamping and 25 control cases; 55.4% clamping) and late (2024–2026: 34 clamping and 28 control cases; 54.8% clamping) study intervals (*p* = 0.954), indicating stable procedural allocation over time rather than late-phase procedural adoption. Patient selection, exclusion criteria, and the group assignment process are summarized in Figure 1.

### 2.2. Surgical Technique and Temporary Uterine Artery Clamping

All patients underwent standard laparoscopic myomectomy under general anesthesia. Patients were placed in the 30° Trendelenburg position, and pneumoperitoneum was created to maintain an intra-abdominal pressure of 14 mmHg. General anesthesia was maintained using a standardized institutional protocol with restrictive crystalloid fluid administration (balanced crystalloids at 5–8 mL/kg/h) to ensure consistent perioperative volume status across both groups. Laparoscopic access was achieved using a 10 mm camera port placed at the umbilicus and three 5 mm auxiliary working ports. Although trocar placement was adapted as necessary based on the size and location of the leiomyomas, the basic surgical approach was applied similarly in all patients.

Once the location of the leiomyoma was determined, an appropriate incision was made in the uterine serosa and myometrium using monopolar scissors. The incision was planned transversely or longitudinally depending on the leiomyoma’s location and was made to a length appropriate for the leiomyoma’s diameter. Vasopressin or any other local vasoconstrictor agent was not used; nor was any additional hemostatic agent, such as a uterine tourniquet or intravenous tranexamic acid, administered. The leiomyoma was enucleated from the pseudocapsular plane by applying traction and counter-traction.

After the leiomyoma was removed, hemostasis was achieved by applying coagulation with bipolar forceps to areas showing bleeding at the leiomyoma site; subsequently, the uterine defect was closed in two layers using the barbed suture technique with the same suture material in all patients. The first layer was applied to close the myometrial defect and reduce the dead space, while the second layer was applied to ensure the anatomical integrity of the uterine wall and serosal surface. After hemostasis was achieved, uterine reconstruction was completed, and the surgical site was re-evaluated for bleeding. During the removal of the excised leiomyomas from the abdominal cavity, closed (in-bag) morcellation was performed in all cases; open morcellation was not used in any patient.

In the clamping arm, bilateral retroperitoneal dissection was attempted prior to myomectomy based on the clamping intention established at initial diagnostic laparoscopic inspection; in cases where dense obliterative adhesions prevented safe vascular access prior to clamp placement, the procedure was converted to laparotomy, recorded as a failed access attempt, and retained in the 134-patient surgical cohort. In completed laparoscopic clamping cases, the ureters were identified and safely retracted laterally, and the uterine arteries were exposed at the level where they arise from the anterior branch of the internal iliac artery. Due to the presence of numerous uterine veins running around the uterine artery, circumferential dissection of the artery using sutures or vascular clips was technically challenging; therefore, laparoscopic Bulldog vascular clamps were preferred because they are easy to apply and remove and carry a low risk of causing trauma to the vessel wall. Only the bilateral uterine arteries were clamped; the utero-ovarian arteries were not included in the uterine artery clamping to preserve ovarian blood flow. The clamps were left in place until the reconstruction of the uterine defect was completed and adequate hemostasis was achieved; they were then removed to restore uterine blood flow. In the control group, laparoscopic myomectomy was performed without temporary clamping of the uterine arteries. The other steps of the surgical procedure were carried out in accordance with standard surgical principles, and all operations were performed by a single surgeon.

### 2.3. Data Collection and Variables Under Study

Patients’ clinical, laboratory, and surgical data were obtained retrospectively from surgical records and patient charts. The data were collected independently by two researchers and cross-checked. The recorded variables included age, body mass index (BMI), number of pregnancies, preoperative hemoglobin level, postoperative hemoglobin level, leiomyoma size, and weight of the resected leiomyoma. Leiomyoma diameter was defined as the maximum dimension of the dominant leiomyoma measured intraoperatively with a graduated laparoscopic probe prior to in-bag morcellation; in cases with multiple leiomyomas, the largest lesion was recorded as the index size. Leiomyoma weight was obtained independently from pathology records and represented the total cumulative weight of all resected myomatous tissue weighed directly on calibrated digital scales in the pathology laboratory, without any mathematical derivation from diameter measurements. The preoperative hemoglobin value was recorded based on the results of a complete blood count (CBC) obtained within 24 h prior to surgery. The postoperative hemoglobin level was assessed based on the results of a blood count performed approximately 24 h after surgery (on postoperative day 1) for routine monitoring. Since patients who received blood transfusions between the preoperative and postoperative hemoglobin measurements were excluded from the study, the hemoglobin levels evaluated were not affected by transfusion.

The primary outcome measures of the study were the absolute hemoglobin decrease and the percentage hemoglobin decrease; the methods for calculating these measures are described in Section 2.4. Operative duration, exact clamping intervals, and estimated blood loss were not systematically available in historical records and therefore could not be evaluated. Operative notes and surgical records were systematically re-reviewed for FIGO anatomical classification, myometrial depth, and vascularity; however, these features were documented exclusively as non-standardized qualitative narrative text rather than discrete, reproducible metrics, precluding their valid inclusion as covariates in multivariable regression models.

### 2.4. Definition of Outcome Measures

The primary outcome measures in this study were defined as the absolute decrease in hemoglobin and the percentage decrease in hemoglobin. Both measures were treated as continuous variables and used as dependent variables in both between-group comparisons and multivariable regression models.

The absolute decrease in hemoglobin was calculated using the following formula:

Absolute hemoglobin decrease (g/dL) = Preoperative hemoglobin − Postoperative hemoglobin

The percentage decrease in hemoglobin was calculated using the preoperative hemoglobin value as a reference in order to account for the effects of individual differences in baseline hemoglobin levels:

Percentage decrease in hemoglobin (%) = [(Preoperative hemoglobin − Postoperative hemoglobin)/Preoperative hemoglobin] × 100

These two measures reflect different aspects of the decrease in hemoglobin. While the absolute decrease in hemoglobin expresses the amount of hemoglobin lost during the perioperative period in g/dL, the percentage decrease in hemoglobin indicates the relative magnitude of this change relative to the baseline hemoglobin level. Because the same amount of blood loss can result in different absolute changes in patients with different baseline hemoglobin levels, the percentage decrease in hemoglobin was also evaluated as a primary outcome measure.

The absolute and percentage decreases in hemoglobin were compared between patients who underwent temporary uterine artery clamping and those who did not; postoperative hemoglobin levels were presented as an additional comparison without adjustment for baseline values. In addition, multivariable models incorporating clinical and leiomyoma-related variables were constructed to assess the adjusted association between uterine artery clamping and the decrease in hemoglobin; the structure of these models and the variable selection are described in detail in Section 2.5.

### 2.5. Statistical Analysis

Statistical analyses were performed using IBM SPSS Statistics version 26. The distribution characteristics of continuous variables were assessed analytically using the Shapiro–Wilk test (with *p* > 0.05 indicating normality) and visually through normal Q-Q plots, histograms, and skewness/kurtosis coefficients. Continuous variables meeting normality criteria (age, BMI, preoperative hemoglobin, postoperative hemoglobin, absolute and percentage hemoglobin declines, leiomyoma size, and leiomyoma weight) were presented as mean ± standard deviation and compared between groups using the independent-samples Student’s t-test. Non-normally distributed discrete variables (gravidity) were reported as median and interquartile range (IQR) and compared using the non-parametric Mann–Whitney U test. Categorical variables were presented as counts and percentages, and procedural safety outcomes (blood transfusion, conversion to laparotomy, and their composite safety outcome) were compared between groups using Fisher’s exact test.

Bivariate relationships among continuous variables were evaluated using Pearson correlation analysis, while Spearman rank correlation was additionally applied for the discrete, non-normally distributed gravidity variable. Two multivariable linear regression models were constructed for absolute hemoglobin decline, simultaneously adjusting for clamping status, preoperative hemoglobin, age, BMI, and tumor burden (Model 1 including dominant diameter and Model 2 including specimen weight). Similarly, two multivariable models were constructed for percentage hemoglobin decline (excluding preoperative hemoglobin to avoid mathematical endogeneity). Multicollinearity was evaluated using variance inflation factors (VIF). In these models, the specification incorporating dominant diameter was pre-specified as the primary analysis on clinical grounds, as dominant diameter is the routinely recorded standard clinical measure of tumor burden; the specification incorporating specimen weight served as a sensitivity analysis, since the two are collinear proxies of tumor burden (r = 0.995). In both the absolute and percentage hemoglobin decline models, the group in which temporary uterine artery clamping was not performed was used as the reference category. Regression results were presented as the regression coefficient (B), 95% confidence interval (95% CI), and *p*-value. Model fit was assessed using R^2^, adjusted R^2^, and the F-statistic. Analyses were performed only on patients with a complete dataset for the variables under study; no missing data imputation was applied. All analyses were conducted two-sided, and *p* < 0.05 was considered statistically significant.

### 2.6. Ethical Approval

The study was conducted in accordance with the ethical principles of the Helsinki Declaration [22] and was approved by the Ethics Committee for Non-Interventional Research in Health Sciences at Bandırma Onyedi Eylül University (Date: 15 June 2026, Decision no.: 2026-06). Due to the study’s retrospective design and the fact that the data were obtained from existing patient records, the ethics committee granted an exemption from the requirement for informed consent. Patient data were analyzed in a de-identified form and stored in accordance with data privacy principles.

## 3. Results

### 3.1. Study Population and Initial Characteristics

Patient disposition and procedural safety outcomes across the 134 surgical patients (65 controls and 69 clamping cases) are summarized in Figure 1. In dedicated safety analyses, allogeneic blood transfusion occurred in 10.8% (7/65) of controls compared with 2.9% (2/69) of patients in the temporary clamping group (Fisher’s exact test *p* = 0.090; Relative Risk [RR] = 0.27, 95% CI: 0.06 to 1.25). Conversion to laparotomy occurred in 7.7% (5/65) of controls and in 2.9% (2/69) of clamping cases (Fisher’s exact test *p* = 0.264; RR = 0.38, 95% CI: 0.08 to 1.88). While procedural complications were numerically lower in the clamping cohort, standalone statistical significance was not reached. However, when evaluated as a composite safety outcome (occurrence of either blood transfusion or conversion to laparotomy across the 134 surgical patients), composite procedural safety events were significantly lower in the temporary clamping cohort (5.8% [4/69] vs. 18.5% [12/65]; Fisher’s exact test *p* = 0.033; Relative Risk = 0.31, 95% CI: 0.11 to 0.92). A total of 118 patients completed laparoscopic surgery without blood transfusion and were included in the continuous hemoglobin decline analyses (53 controls and 65 clamping cases). When the distribution of baseline characteristics between the two groups was examined, the mean age of patients who underwent temporary uterine artery clamping was 33.46 ± 7.11 years, while that of patients who did not undergo the procedure was 33.23 ± 7.27 years. The BMI was 26.62 ± 1.58 kg/m^2^ in the clamping group and 26.72 ± 1.57 kg/m^2^ in the non-clamping group. The number of pregnancies was recorded as a median of 1 (interquartile range: 2) in both groups. The preoperative hemoglobin level was 11.84 ± 1.67 g/dL in the clamping group and 11.80 ± 1.60 g/dL in the non-clamping group. The size of the leiomyomas was measured at 6.94 ± 0.68 cm and 6.96 ± 0.69 cm, respectively, while the weight of the resected leiomyomas was 187.82 ± 61.50 g and 189.11 ± 62.10 g, respectively. No cases of ureteral injury, major retroperitoneal vascular trauma, broad ligament hematoma, or unplanned reoperation occurred in either cohort during the perioperative stay.

No significant differences were observed between the groups in terms of age, BMI, number of pregnancies, preoperative hemoglobin level, leiomyoma size, or leiomyoma weight (all *p* > 0.05). The balanced distribution of baseline variables across the groups indicates that the two groups are comparable in terms of perioperative hemoglobin changes. The patients’ baseline clinical, laboratory, and leiomyoma characteristics are presented in Table 1.

### 3.2. Perioperative Hemoglobin Results

Perioperative hemoglobin results are presented in Table 2. Although postoperative hemoglobin levels were numerically higher in the group that underwent temporary uterine artery clamping compared to the group that did not, this difference was not statistically significant (9.59 ± 1.70 vs. 10.01 ± 1.45 g/dL, *p* = 0.153). In contrast, the absolute decrease in hemoglobin in patients who underwent temporary uterine artery clamping was statistically significantly lower than in those who did not undergo the procedure (1.83 ± 0.88 vs. 2.20 ± 0.90 g/dL, *p* = 0.027). Similarly, the percentage decrease in hemoglobin was also significantly lower in the group that underwent clamping (15.24 ± 6.61% vs. 18.84 ± 7.77%, *p* = 0.008).

### 3.3. Correlations Between Clinical Variables and Those Associated with Leiomyoma

**Figure 2:**
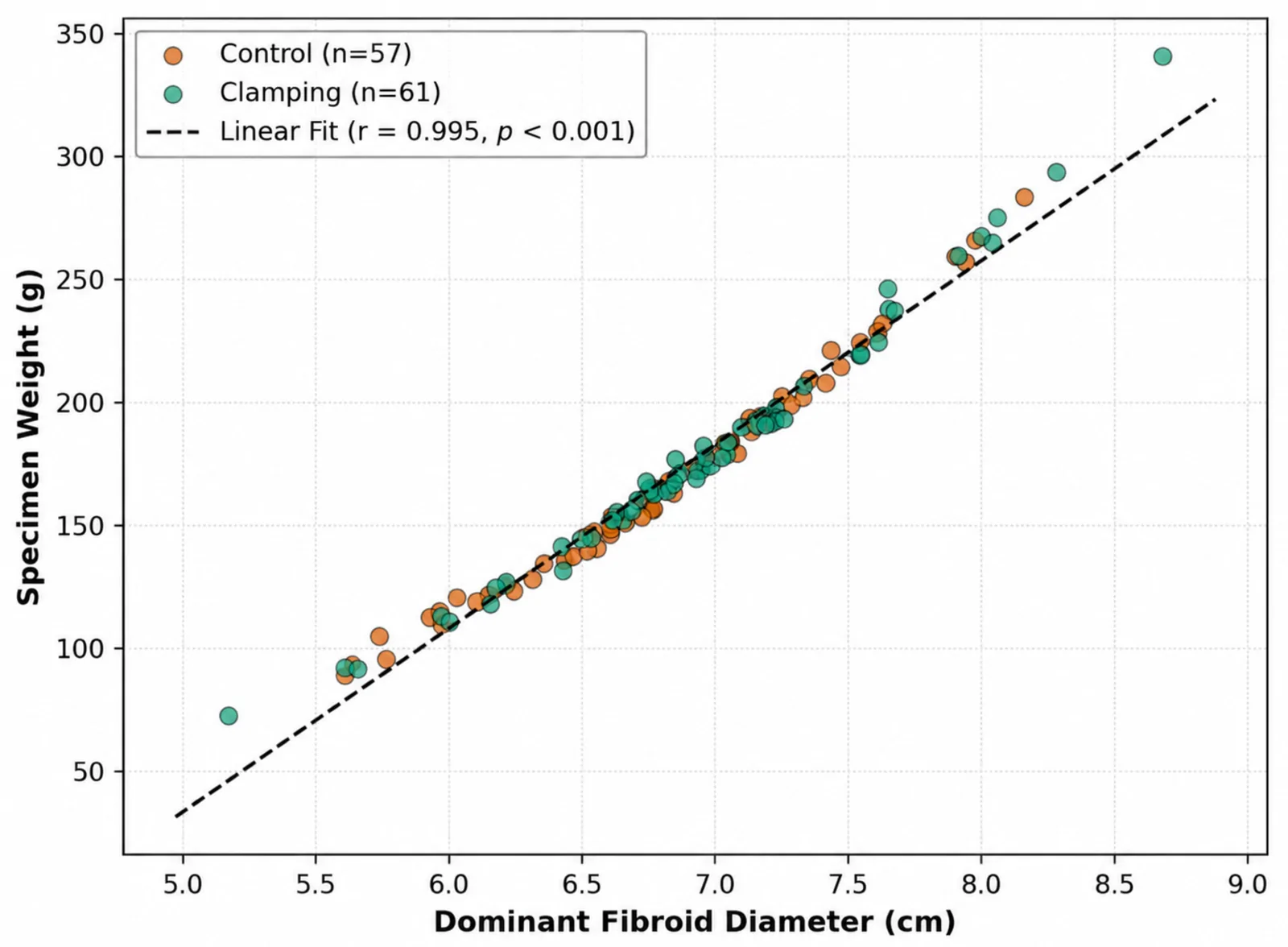
Scatterplot displaying the linear correlation between dominant fibroid diameter (cm) and surgical specimen weight (g) across the study cohort (*n* = 118 complete data pairs), stratified by surgical group (Control vs. Clamping), with fitted linear regression trendline (Pearson r = 0.995, *p* < 0.001).

Pearson correlation analysis revealed a strong positive correlation between age and gravidity (r = 0.820, *p* < 0.001). The strongest correlation was observed between leiomyoma size and leiomyoma weight (r = 0.995, *p* < 0.001; Figure 2). As illustrated in the scatterplot (Figure 2), this near-perfect collinearity reflects the targeted removal of solitary dominant fibroids accounting for nearly all excised tissue mass. Body mass index was not significantly correlated with any of the other parameters examined, and neither age nor gravidity was significantly correlated with leiomyoma size or weight (all *p* > 0.05).

Preoperative hemoglobin was moderately correlated with absolute hemoglobin decline (r = 0.338, *p* < 0.001), while absolute hemoglobin decline was very strongly correlated with percentage hemoglobin decline (r = 0.947, *p* < 0.001). Age, BMI, gravidity, leiomyoma size, and leiomyoma weight were not significantly correlated with either absolute or percentage hemoglobin decline (all *p* > 0.05). No other statistically significant correlations were observed. The bivariate correlation matrix is presented in Table 3.

### 3.4. Multivariable Models for Absolute Hemoglobin Decline

Two multivariable linear regression models were fitted to assess the association between temporary uterine artery clamping and absolute perioperative hemoglobin decline. Both models included clamping status, preoperative hemoglobin, age, and BMI. Myoma size was included in Model 1 and myoma weight in Model 2; these two variables were assessed separately because of their very strong correlation (r = 0.995).

Temporary uterine artery clamping was associated with a lower absolute hemoglobin decline in both models. After adjustment for the other variables, clamping was associated with a 0.383 g/dL lower decline in Model 1 (95% CI: −0.692 to −0.074; *p* = 0.015) and a 0.381 g/dL lower decline in Model 2 (95% CI: −0.690 to −0.073; *p* = 0.016).

Preoperative hemoglobin was positively associated with absolute hemoglobin decline in both models (B = 0.193; 95% CI: 0.099 to 0.288; *p* < 0.001). Age, BMI, myoma size, and myoma weight were not significantly associated with absolute hemoglobin decline in their respective models (all *p* > 0.05).

Both models were statistically significant overall, with R^2^ values of 0.177 (adjusted R^2^ = 0.141, F (5, 112) = 4.827, *p* < 0.001) in Model 1 and 0.180 (adjusted R^2^ = 0.143, F (5, 112) = 4.917, *p* < 0.001) in Model 2. VIF values were close to 1 in both models, indicating no evidence of problematic multicollinearity. The regression results are presented in Table 4.

### 3.5. Multivariable Models for Percentage Decreases in Hemoglobin

Two multivariable linear regression models were fitted to assess the association between temporary uterine artery clamping and percentage perioperative hemoglobin decline. Both models included clamping status, age, and BMI. Myoma size was included in Model 1 and myoma weight in Model 2; these variables were assessed separately because of their very strong correlation (r = 0.995). Preoperative hemoglobin was deliberately not included as a covariate in these models because it is mathematically embedded in the definition of the outcome (percentage decline is calculated using preoperative hemoglobin in its denominator); entering it as a predictor would therefore introduce part-whole endogeneity.

Temporary uterine artery clamping was associated with a lower percentage hemoglobin decline in both models. After adjustment for the other variables, clamping was associated with a 3.619 percentage-point lower decline in Model 1 (95% CI: −6.247 to −0.990; *p* = 0.007) and a 3.601 percentage-point lower decline in Model 2 (95% CI: −6.224 to −0.978; *p* = 0.008). Age, BMI, myoma size, and myoma weight were not significantly associated with percentage hemoglobin decline in their respective models (all *p* > 0.05).

Both models were statistically significant overall (Model 1: F (4, 113) = 2.534, *p* = 0.044; Model 2: F (4, 113) = 2.668, *p* = 0.036), although their explanatory power was limited, with adjusted R^2^ values of 0.050 and 0.054, respectively. VIF values were close to 1 in both models, indicating no evidence of problematic multicollinearity. The regression results are presented in Table 5.

## 4. Discussion

In this retrospective, single-center, comparative observational study, temporary uterine artery clamping performed during laparoscopic myomectomy demonstrated a significant multivariable adjusted association with a reduction in both the absolute and percentage decreases in hemoglobin levels. In patients who underwent clamping, the absolute hemoglobin drop was approximately 0.38 g/dL lower, and the percentage hemoglobin drop was approximately 3.6 percentage points lower; for both outcome measures, the 95% confidence intervals for clamping remained entirely below zero in both multivariable models. In contrast, neither age, BMI, leiomyoma size, nor leiomyoma weight showed an independent association with either of the two outcome measures in their respective models. Conversely, postoperative hemoglobin level alone did not show a significant difference between the two groups. The two groups were similar in terms of baseline clinical, laboratory, and leiomyoma characteristics; preoperative hemoglobin levels were also nearly identical (11.84 ± 1.67 vs. 11.80 ± 1.60 g/dL). Therefore, the observed difference did not stem from an imbalance in the distribution of preoperative anemia. These findings suggest that temporary clamping of the uterine arteries may be a vascular control strategy that could help limit perioperative blood loss during laparoscopic myomectomy.

### 4.1. Initial Characteristics and Mutual Correlations

In our re-audited bivariate analyses, strong and statistically significant correlations were observed strictly where biologically or anatomically expected: between age and gravidity (r = 0.820, *p* < 0.001), and between dominant leiomyoma diameter and cumulative specimen weight (r = 0.995, *p* < 0.001). While epidemiological and endocrine studies emphasize that patient age, parity, and metabolic habitus represent relevant clinical cofactors in leiomyoma pathogenesis [23], and that aging and obesity promote leiomyoma formation and growth through estrogen-mediated mechanisms [24], in our surgical cohort, patient age, BMI, and leiomyoma burden were mutually independent (all *p* > 0.05), and none of these clinical confounders demonstrated a significant correlation with perioperative hemoglobin decline. The very strong correlation between dominant diameter and specimen weight (r = 0.995, *p* < 0.001) reflects two factors. First, in the vast majority of patients in this reproductive-aged cohort, surgery targeted a solitary dominant symptomatic lesion that accounted for nearly all excised tissue mass. Second, although leiomyoma volume scales with the cube of its linear dimension, across the relatively narrow size range of this cohort (6.95 ± 0.68 cm) this cubic relationship is closely approximated by a linear function, so that the Pearson correlation coefficient naturally approaches unity. Because leiomyoma diameter and specimen weight reflect collinear proxies of tumor burden (r = 0.995), evaluating them simultaneously in the same multivariable model would introduce severe multicollinearity. Therefore, they were appropriately evaluated in separate multivariable models (Model 1 with diameter, and Model 2 with weight). Crucially, because patient age, BMI, and baseline hemoglobin were mutually independent, they could be entered simultaneously into each multivariable model without collinearity inflation (all VIF < 1.04). The fact that temporary uterine artery clamping maintained a statistically significant adjusted protective association with consistent effect sizes across both unified models (B = −0.383 g/dL in Model 1 and B = −0.381 g/dL in Model 2) confirms that the observed adjusted association is consistent and not confounded by patient demographics or tumor burden.

### 4.2. Evaluation of the Findings in the Context of the Literature

Our findings are generally consistent with previous studies reporting the potential benefit of uterine artery occlusion for bleeding control during myomectomy, as well as with current guideline recommendations. A systematic review and meta-analysis reported that uterine artery occlusion is associated with lower estimated blood loss, a lower transfusion rate, and a more limited postoperative decline in hemoglobin [25]. Similarly, a meta-analysis of randomized controlled trials reported that temporary bilateral uterine and utero-ovarian artery occlusion reduces operative blood loss and the drop in hemoglobin, shortens the length of hospital stay, but does not significantly alter the duration of surgery [26]. This view is also reflected in a recent American Association of Gynecologic Laparoscopists (AAGL) practice guideline on reducing blood loss during laparoscopic myomectomy, which states that uterine artery occlusion is among the intraoperative interventions supported by moderate-quality evidence for reducing blood loss [14]. In addition, a recently published systematic review and meta-analysis on temporary uterine artery ligation reported, in an analysis encompassing seven studies, a reduction of approximately 85 mL in estimated blood loss and a decrease of approximately 0.4 g/dL in postoperative hemoglobin levels [27]. A study with a retrospective case–control design also demonstrated that temporary uterine artery ligation reduces intraoperative blood loss [28]. However, in a randomized controlled trial, although temporary occlusion of the bilateral uterine and utero-ovarian arteries reduced estimated blood loss, no significant improvement was observed in the difference in preoperative and postoperative hemoglobin levels [29]. The differences among these studies may be related to heterogeneity in patient selection, the number and size of leiomyomas, the extent of occluded vascular structures, clamping technique, perioperative fluid management, the timing of hemoglobin measurements, and methods for assessing blood loss. These variable results in the previous literature highlight the importance of evaluating the effect of uterine artery clamping not only based on estimated blood loss but also through standardized perioperative hematological variables.

### 4.3. Possible Mechanism and Safety Assessment

The potential effect of temporary uterine artery clamping can also be explained from both anatomical and physiological perspectives. Temporary clamping of both uterine arteries may limit bleeding in the surgical field by reducing arterial blood flow to the uterus and local perfusion pressure during leiomyoma enucleation and myometrial reconstruction [30]. This effect is particularly important during procedures with a high potential for bleeding, such as myometrial incision, enucleation, and closure of the uterine defect. In our study, the uterine arteries were exposed retroperitoneally and temporarily clamped with bilateral laparoscopic bulldog clamps at the level where they arise from the anterior branch of the internal iliac artery; after uterine reconstruction was completed, the clamps were removed to restore uterine perfusion. Previous studies using similar temporary clamping techniques have demonstrated that this approach can reduce perioperative blood loss and the need for transfusions [31]. In contrast, a randomized controlled trial with a two-year follow-up that evaluated permanent uterine artery occlusion reported that the procedure was performed without adversely affecting markers of ovarian reserve [32].

Intramyometrial vasopressin injection is often considered the pharmacological gold standard for bleeding management in the surgical field, and its effectiveness in reducing surgical blood loss has been demonstrated in the current literature [18,33]. However, the fact that vasopressin was not used in any of the patients in our study allowed us to evaluate the hemostatic efficacy of temporary uterine artery clamping in its pure form, without any pharmacological confounders. Given the risks of systemic hypertension, bradycardia, and—albeit rarely—serious cardiovascular complications associated with the use of vasopressin [33], our retroperitoneal temporary clamping technique offers a feasible alternative that provides mechanical and reversible vascular control without creating a hemodynamic burden.

Another critical clinical concern regarding uterine vascular control techniques is the potential for transient ischemia to impair ovarian perfusion via utero-ovarian anastomoses, thereby compromising ovarian reserve. However, the current literature has shown that in cases where temporary uterine artery occlusion is performed, postoperative AMH levels and antral follicle counts are preserved, and ovarian reserve is not adversely affected in the long term [34]. Crucially, postoperative AMH levels, antral follicle counts, and direct measures of ovarian or uterine tissue perfusion were not evaluated in our cohort; therefore, no direct conclusions regarding ovarian reserve preservation or organ perfusion can be drawn from our data. Any inference regarding reproductive safety relies strictly on external published literature rather than findings obtained in the present study. Importantly, our findings reflect only immediate 24 h hematological outcomes and cannot be extrapolated to long-term uterine integrity, myometrial healing, or subsequent obstetric performance. Uterine rupture during pregnancy remains a documented, devastating complication following laparoscopic myomectomy [35], underscoring that transient uterine devascularization requires rigorous long-term obstetric surveillance.

Finally, the safety of specimen extraction must be conceptually and procedurally decoupled from the vascular safety of temporary uterine artery clamping. In our cohort, contained in-bag morcellation was systematically performed in all cases to eliminate the risk of inadvertent intraperitoneal tissue fragmentation. Limiting tissue fragment and cellular dispersal within the peritoneal cavity is clinically paramount; uncontained morcellation carries documented risks of delayed iatrogenic complications, notably disseminated peritoneal leiomyomatosis (DPL) and parasitic leiomyomas [36], alongside the catastrophic risk of dispersing unanticipated uterine leiomyosarcoma. While endobag containment provides essential mechanical and oncological protection during tissue retrieval, temporary uterine artery clamping functions strictly as a reversible hemodynamic intervention to reduce intraoperative bleeding; the safety profiles and surgical objectives of these two procedural steps are fundamentally distinct.

### 4.4. Perioperative Hemoglobin Measures, Effect Size, and Multivariable Adjusted Association

One of the contributions of our study to the literature is that perioperative blood loss was assessed not only by postoperative hemoglobin concentration but also by both the absolute and percentage decreases in hemoglobin. A single postoperative hemoglobin value may be influenced by the patient’s baseline hemoglobin level, perioperative fluid replacement, and the time of blood sample collection. Therefore, the change in hemoglobin compared to the patient’s own preoperative value allows for a more comprehensive assessment of perioperative hematological loss. In our study, while the postoperative hemoglobin concentration was numerically higher in the clamping group—though not statistically significant—both the absolute and percentage decreases in hemoglobin were found to be significantly lower. This suggests that focusing solely on postoperative hemoglobin levels may overlook the more limited but consistent hematological differences between vascular control strategies [37].

In multivariable analyses, uterine artery clamping maintained a significant adjusted association with a lower absolute (−0.38 g/dL) and percentage (−3.6 percentage points) decrease in hemoglobin. However, this statistical adjustment does not eliminate the substantial risk of confounding by indication. Because clamping was selectively undertaken in cases with perceived hypervascularity, deep intramural location, or anticipated reconstructive difficulty, unmeasured anatomical factors omitted from the models may have influenced both surgical group allocation and operative blood loss. The magnitude of this effect is comparable to the difference in hemoglobin levels reported in meta-analytic data on temporary uterine artery ligation [27]. However, when blood transfusions and conversions to laparotomy were evaluated together as a composite major procedural safety endpoint across all 134 surgical patients, temporary uterine artery clamping was associated with a statistically significant reduction in composite morbidity (5.8% vs. 18.5%, *p* = 0.033; Relative Risk = 0.31, 95% CI: 0.11 to 0.92), underscoring its overall clinical safety benefit. Nevertheless, as noted, the clinical magnitude of a 0.38 g/dL preservation in hemoglobin decline remains modest in absolute terms and did not translate into a statistically significant reduction in standalone transfusion rates (10.8% vs. 2.9%, *p* = 0.090). Therefore, temporary clamping should be viewed not as a definitive intervention that eliminates clinical anemia on its own, but rather as an adjunctive surgical measure that provides modest perioperative hematological stabilization in selected cases.

It is noteworthy that in models examining the absolute decrease in hemoglobin, the preoperative hemoglobin level is also an independent and positive predictor. The observation that the absolute decrease is greater in patients with higher baseline hemoglobin levels may be related to the fact that the same amount of perioperative blood loss can result in different absolute changes depending on the baseline hematological status, and that perioperative fluid balance can affect the measured hemoglobin concentration [38]. This finding also highlights some of the limitations of using the absolute hemoglobin difference on its own [39]. For this reason, the separate analysis of the percentage decrease in hemoglobin is an important methodological aspect of the study. Since the percentage change is a measure that directly includes the preoperative hemoglobin level, this variable was not included as a separate covariate in the regression models for the percentage decrease in hemoglobin; nevertheless, uterine artery clamping remained statistically significant in all models.

The fact that leiomyoma size and weight do not show an independent association with absolute or percentage decreases in hemoglobin in our regression models should be interpreted with caution in light of the current literature. The current literature emphasizes that total fibroid volume (cm^3^), calculated using three-dimensional ultrasound or magnetic resonance imaging, and tumor vascularization are key predictors of surgical blood loss and hemoglobin decline [10]. The fact that leiomyoma size and weight did not emerge as independent predictors in our study should not be interpreted to mean that leiomyoma burden has no effect on bleeding. This finding can be explained by several methodological and clinical factors. First, the distribution of leiomyoma size in our patient population is relatively homogeneous; this narrow size range may have limited the statistical discriminatory power of the regression models. Second, a unidimensional linear diameter measurement or the weight of the excised specimen may be insufficient to fully reflect the tumor’s three-dimensional volume and the extent of the myometrial vascular bed. Third, and most importantly, the strong hemostatic suppression provided by the temporary uterine artery clamping we performed may have masked or prevented bleeding differences that could have arisen based on myoma size within the examined size range. Indeed, the literature has demonstrated the combined effect of anatomical factors—such as leiomyoma size, FIGO classification, intramural depth of invasion, and degree of vascularization—on blood loss [8,11,40]. Therefore, our findings suggest that, when effective vascular control is achieved, the decrease in hemoglobin in leiomyomas within the standard size range is influenced more by the hemostatic technique used than by tumor size.

### 4.5. Strengths and Limitations

Certain methodological aspects of the study support the reliability of the findings. The fact that all surgeries were performed by the same surgeon, that the basic laparoscopic approach was consistent, that no patient received vasopressin or any other local vasoconstrictor, and that the uterine defect was closed in all patients using a double-layer barbed suture technique with the same suture material reduced variability that might have arisen from the surgical technique. Furthermore, assessing preoperative hemoglobin within 24 h prior to surgery and postoperative hemoglobin at 24 h postoperatively—once perioperative fluid balance and hemodilution had stabilized—contributed to the standardization of measurement timing and a more objective reflection of actual blood loss. Other strengths of the study include the similarity between the clamping and non-clamping groups in terms of age, BMI, gravidity, preoperative hemoglobin, and leiomyoma size and weight, as well as the testing of the association using various multivariable models.

However, this study has some limitations. First, due to its retrospective design, our findings represent an adjusted association rather than a causal relationship. Confounding by indication remains a primary methodological limitation; the intraoperative criteria that guided clamping (pronounced surface vascularity, deep intramural location, and anticipated reconstructive difficulty) were recorded qualitatively in historical records and could not be standardized into numerical variables for regression adjustment. Consequently, adjusting for age, BMI, dominant diameter, and specimen weight cannot exclude residual confounding related to fibroid vascularity and tissue architecture. Unmeasured anatomical features influencing the decision to clamp may account for part of the observed attenuation in hemoglobin decline; thus, findings should be interpreted with appropriate caution. In addition, patients who required perioperative blood transfusion or conversion to laparotomy were excluded from the continuous hemoglobin decline models. Although these clinical outcomes were analyzed separately in the surgical cohort, post-intervention exclusions inherently carry a risk of selection bias; because the net direction of this bias cannot be definitively ascertained from observational event counts alone, this remains an acknowledged methodological limitation. Second, while the single-center, single-surgeon design standardizes the operative approach, subtle influences of the surgical learning curve across the five-year study period cannot be completely ruled out. Additional sensitivity analyses incorporating operative period (2021–2023 vs. 2024–2026) into the primary diameter-based models yielded similar estimates for uterine artery clamping. Clamping remained associated with lower absolute hemoglobin decline (B = −0.387 g/dL; 95% CI: −0.695 to −0.080; *p* = 0.014) and lower percentage hemoglobin decline (B = −3.656 percentage points; 95% CI: −6.266 to −1.046; *p* = 0.006). Operative period was not significantly associated with either outcome (absolute decline: B = −0.235 g/dL; 95% CI: −0.555 to 0.085; *p* = 0.148; percentage decline: B = −2.245 percentage points; 95% CI: −4.959 to 0.469; *p* = 0.104). Nevertheless, minor unmeasured refinements in surgical efficiency or perioperative care over time cannot be excluded. Furthermore, conducting the study at a single specialized center inherently limits the external generalizability of our findings to other hospitals across Türkiye and international healthcare settings, and inter-operator variability across different surgical skill levels could not be evaluated. In addition, the final sample of 118 ASA Physical Status I–II patients reflects predefined baseline eligibility criteria (such as exclusion of multi-surgeon procedures and concurrent pelvic interventions) together with restriction of the continuous hemoglobin models to uncomplicated, completed laparoscopic cases, which was undertaken to avoid transfusion-related confounding of postoperative hemoglobin; conversions and transfusions were separately evaluated as safety endpoints in the 134-patient surgical cohort. This restriction improves internal validity but limits generalizability to the full clinical spectrum and to other regional practice patterns. Third, our data archive does not contain direct information on variables such as estimated intraoperative blood loss, operative time, and clamping duration. A limitation is that operative time and exact clamping duration could not be evaluated due to the retrospective nature of surgical records. Bilateral retroperitoneal dissection inevitably prolongs operative duration, and because operative time was not documented, whether this additional surgical step is counterbalanced by improved operative visualization remains unproven. Furthermore, while immediate complications such as ureteral or major vascular injury were absent, the lack of recorded ischemic duration and long-term obstetric follow-up precludes definitive claims regarding comprehensive procedural safety. Furthermore, the retrospective design precluded systematic recording of FIGO classification, intramural depth, and the exact number or localization of myomas; although dominant diameter and aggregate specimen weight were controlled for, residual confounding related to fibroid architecture cannot be completely excluded. Additionally, perioperative changes in hemoglobin levels are an indirect surrogate of blood loss and may be modulated by intraoperative fluid administration and hemodilution; given the modest adjusted difference of 0.38 g/dL, these findings should not be interpreted as a direct measure of actual volumetric blood loss. In addition, our dataset was restricted to the immediate perioperative period; long-term obstetric outcomes, pregnancy rates, and potential scar complications in subsequent pregnancies were not tracked. Fourth, an a priori sample size calculation was not performed due to the retrospective consecutive enrollment design. Evaluating estimation precision through 95% confidence intervals demonstrates that while multivariable models for dynamic decline parameters excluded the null (absolute decline 95% CI: −0.692 to −0.074 g/dL; percentage decline 95% CI: −6.247 to −0.990 percentage points), the static postoperative hemoglobin comparison (mean difference 0.42 g/dL, 95% CI: −0.15 to 0.99 g/dL; *p* = 0.153) was insufficiently precise to distinguish between true clinical equivalence and potential differences up to approximately 1.0 g/dL. Crucially, a non-significant *p*-value cannot be interpreted as proof that no difference exists; rather, it indicates that wider confidence intervals stemming from our sample size cannot rule out modest hematological variations, underscoring the necessity of larger prospective trials.

Finally, the modest coefficient of determination in our regression models (R^2^ = 0.180 for absolute decline and R^2^ = 0.086 for percentage decline) reflects the complex multifactorial nature of perioperative blood loss. In laparoscopic myomectomy, unmeasured physiological variables—most notably HBL into the retroperitoneum and myometrial dead space [41], individual microvascular density, and dynamic fluid shifts account for substantial residual hematological variance that cannot be captured by standard patient demographics. Nevertheless, the adjusted association of temporary uterine artery clamping in attenuating perioperative hemoglobin decline maintained its statistical significance and directional consistency across all adjusted models.

## 5. Conclusions

In conclusion, temporary uterine artery clamping demonstrated a statistically significant adjusted association with a modest attenuation in absolute (0.38 g/dL) and percentage (3.6 percentage points) hemoglobin decline during laparoscopic myomectomy, whereas static postoperative hemoglobin levels alone did not differ significantly between groups, supporting the value of baseline-referenced dynamic parameters in assessing perioperative blood loss. Given the modest clinical magnitude of this difference and the potential for residual confounding by indication stemming from unmeasured intraoperative anatomical complexity, temporary clamping should be considered a feasible adjunctive strategy in experienced hands rather than a definitive routine intervention. Prospective, multicenter randomized controlled trials with standardized recording of anatomical characteristics, operative duration, ischemic clamping times, and long-term reproductive outcomes are required to validate these findings and establish broader clinical safety.

## Figures and Tables

**Table 1:** A Comparison of the Study Groups in Terms of Baseline Clinical, Laboratory, and Leiomyoma Characteristics.

Variable	No Clamping (*n* = 53)	Temporary Uterine Artery Clamping (*n* = 65)	*p* Value
Age, years	33.23 ± 7.27	33.46 ± 7.11	0.860
Body mass index, kg/m^2^	26.72 ± 1.57	26.62 ± 1.58	0.733
Gravidity, median (IQR)	1 (2)	1 (2)	0.909
Preoperative hemoglobin, g/dL	11.80 ± 1.60	11.84 ± 1.67	0.877
Myoma size, cm	6.96 ± 0.69	6.94 ± 0.68	0.865
Myoma weight, g	189.11 ± 62.10	187.82 ± 61.50	0.910

Note: Data are presented as mean ± standard deviation unless otherwise indicated. Gravidity is presented as median (interquartile range) and was compared using the Mann–Whitney U test; the remaining continuous variables (age, body mass index, preoperative hemoglobin, myoma size, and myoma weight) were compared using the independent-samples *t* test. Two-sided *p* values < 0.05 were considered statistically significant. *n*, number of patients; SD, standard deviation; IQR, interquartile range.

**Table 2:** Comparison of the postoperative hemoglobin levels, absolute hemoglobin decline, and percentage hemoglobin decline among the study groups.

Variable	No Clamping (*n* = 53)	Temporary Uterine Artery Clamping (*n* = 65)	*p* Value
Postoperative hemoglobin, g/dL	9.59 ± 1.70	10.01 ± 1.45	0.153
Absolute hemoglobin decline, g/dL	2.20 ± 0.90	1.83 ± 0.88	**0.027**
Percentage hemoglobin decline, %	18.84 ± 7.77	15.24 ± 6.61	**0.008**

Note: Data are presented as mean ± standard deviation. Groups were compared using the independent-samples *t* test. Absolute hemoglobin decline was defined as preoperative hemoglobin minus postoperative hemoglobin, and percentage hemoglobin decline as [(preoperative hemoglobin − postoperative hemoglobin)/preoperative hemoglobin] × 100. Two-sided *p* values < 0.05 were considered statistically significant and are shown in bold. *n*, number of patients; SD, standard deviation.

**Table 3:** Bivariate correlation matrix of demographic, clinical, and leiomyoma characteristics (*n* = 118).

Variable	1. Age	2. BMI	3. Gravidity	4. Preop Hb	5. Myoma Size	6. Absolute Hb Decline	7. Myoma Weight	8. Hb Decline (%)
1. Age	—							
2. Body mass index	−0.153	—						
3. Gravidity	0.820 **	−0.127	—					
4. Preoperative Hb	−0.057	0.029	−0.057	—				
5. Myoma size	0.041	−0.112	0.104	0.014	—			
6. Absolute Hb decline	0.028	−0.115	0.037	0.338 **	−0.028	—		
7. Myoma weight	0.037	−0.104	0.102	0.015	0.995 **	−0.055	—	
8. Hb decline (%)	0.046	−0.125	0.063	0.041	−0.058	0.947 **	−0.086	—

Note: Values represent bivariate pairwise Pearson correlation coefficients (r). For the non-parametric count variable (gravidity), Spearman rank correlation analysis yielded fully concordant results (rho = 0.820 with age, *p* < 0.001; and no significant correlation with other parameters). ** Correlation is significant at the 0.01 level (2-tailed, unadjusted). BMI, body mass index; Hb, hemoglobin; %, percentage; Preop, preoperative.

**Table 4:** Multivariable linear regression models for absolute hemoglobin decline (*n* = 118).

Variable	Model 1: B (95% CI)	*p*	Model 2: B (95% CI)	*p*
Uterine artery clamping	−0.383 (−0.692 to −0.074)	**0.015**	−0.381 (−0.690 to −0.073)	**0.016**
Preoperative hemoglobin, g/dL	0.193 (0.099 to 0.288)	**<0.001**	0.193 (0.099 to 0.288)	**<0.001**
Age, years	0.004 (−0.018 to 0.026)	0.705	0.004 (−0.018 to 0.026)	0.701
BMI, kg/m^2^	−0.074 (−0.173 to 0.025)	0.142	−0.075 (−0.174 to 0.024)	0.134
Myoma size, cm	−0.056 (−0.285 to 0.174)	0.632	—	—
Myoma weight, g	—	—	−0.001 (−0.004 to 0.002)	0.439

Model 1: R^2^ = 0.177, adjusted R^2^ = 0.141, F(5, 112) = 4.827, *p* < 0.001. Model 2: R^2^ = 0.180, adjusted R^2^ = 0.143, F(5, 112) = 4.917, *p* < 0.001. Note: Both models included uterine artery clamping status, preoperative hemoglobin, age, and body mass index. Model 1 represents the primary model incorporating dominant diameter, whereas Model 2 represents a sensitivity analysis substituting specimen weight, to confirm that the estimates are robust to the choice of tumor-burden metric (r = 0.995). Both models included 118 patients. VIF values ranged from 1.002 to 1.037 in Model 1 and from 1.002 to 1.035 in Model 2. Bold values indicate *p* < 0.05. B, unstandardized regression coefficient; CI, confidence interval; BMI, body mass index; VIF, variance inflation factor.

**Table 5:** Multivariable linear regression models for percentage hemoglobin decline (*n* = 118).

Variable	Model 1 B (95% CI)	*p*	Model 2 B (95% CI)	*p*
Uterine artery clamping	−3.619 (−6.247 to −0.990)	**0.007**	−3.601 (−6.224 to −0.978)	**0.008**
Age, years	0.034 (−0.152 to 0.219)	0.719	0.034 (−0.151 to 0.219)	0.716
BMI, kg/m^2^	−0.617 (−1.461 to 0.227)	0.151	−0.626 (−1.468 to 0.215)	0.143
Myoma size, cm	−0.719 (−2.671 to 1.233)	0.467	—	—
Myoma weight, g	—	—	−0.011 (−0.032 to 0.010)	0.312

Model 1: R^2^ = 0.082, adjusted R^2^ = 0.050, F (4, 113) = 2.534, *p* = 0.044. Model 2: R^2^ = 0.086, adjusted R^2^ = 0.054, F (4, 113) = 2.668, *p* = 0.036. Note: Both models included uterine artery clamping status, age, and body mass index. Model 1 represents the primary model incorporating dominant diameter, whereas Model 2 represents a sensitivity analysis substituting specimen weight, to confirm that the estimates are robust to the choice of tumor-burden metric (r = 0.995). Preoperative hemoglobin was not included in these models to avoid part-whole endogeneity. Both models included 118 patients. Regression coefficients represent changes in percentage points. VIF values ranged from 1.002 to 1.036 in Model 1 and from 1.002 to 1.035 in Model 2. Bold values indicate *p* < 0.05. B, unstandardized regression coefficient; CI, confidence interval; BMI, body mass index; VIF, variance inflation factor.

## Data Availability

The original contributions presented in this study are included in the article. Further inquiries can be directed to the corresponding author.

## Abbreviations

AAGL	American Association of Gynecologic Laparoscopists
AMH	Anti-Müllerian hormone
BMI	Body mass index
CBC	Complete blood count
CI	Confidence interval
FIGO	International Federation of Gynecology and Obstetrics
FSH	Follicle-stimulating hormone
HBL	Hidden blood loss
IQR	Interquartile range
SD	Standard deviation
SPSS	Statistical Package for the Social Sciences
STROBE	Strengthening the Reporting of Observational Studies in Epidemiology
ASA	American Society of Anesthesiologists
RR	Relative risk
VIF	Variance inflation factor
